# Supplementary material for: Analysis of circulating-microRNA expression in lactating Holstein cows under summer heat stress
Source: PLoS One. 2020 Aug 31;15(8):e0231125. doi: 10.1371/journal.pone.0231125 (PMC7458322; doi:10.1371/journal.pone.0231125)
Supplement: Attachment — Submitted filename: PLOS.docx [file pone.0231125.s003.docx]

The manuscript entitled “Analysis of circulating-microRNA expression in lactating Holstein cows under summer heat stress” investigates the changes in circulatory (blood) microRNAs in response to heat stress in pregnant and non-pregnant lactating dairy cows. Although it is an attractive subject, some methodological issues need to be clarified.

Please explain in more detail: how many blood samples have been taken from the cows? Was there only one measurement per cow in spring and one in summer? Please specify when measurements were taken- what month and day were chosen?

As parameter of exposure to heat was chosen THI in barn. Were cows at any time exposed to the outside temperatures?

Pregnancy as a matter of investigation: were all pregnant cows of the same gestational age?

In the present model of investigation where comparison of blood samples of the same pregnant cows but in different time period (and different gestational age) were made in order to investigate factor-heat stress could be disputable. Namely, differences observed in different time periods could be related to pregnancy state and not heat stress. Differences in progesterone levels observed also, could be related to the stage of pregnancy and not necessary induced by heat stress.

Line 105: the authors stated: “Except for two cows, all cows were similar…” Were these differences taken into account considering results analysis?

Results: Statistical significances were not labeled neither in Table 1 nor in Figures

Line 224: it is not stated under what conditions significance was observed

Line 237: please rephrase, it is not clear at what time point significant difference was observed and please explain …”and milk increased after HS”? compared to what? That’s not what Figure shows.
