# Supplementary material for: Analysis of circulating-microRNA expression in lactating Holstein cows under summer heat stress
Source: PLoS One. 2020 Aug 31;15(8):e0231125. doi: 10.1371/journal.pone.0231125 (PMC7458322; doi:10.1371/journal.pone.0231125)
Supplement: Attachment — Submitted filename: Response to reviewers 1.docx [file pone.0231125.s004.docx]

Response to general comments

1.    Please ensure that your manuscript meets PLOS ONE's style requirements, including those for file naming.

***Re: According to the PLOS ONE’s style, we checked and rewrote.***

2. In your Methods section, please provide additional details regarding the animals used in your study and ensure you have described the source.

***Re: we provided additional information. Pls see the responses to reviewer 1 comments***

3. In your Methods section, please state the volume of the blood samples collected for use in your study.

***Re: we described. Pls see the responses to reviewer 1 comments***

4. In your Methods section, please include a comment about the state of the animals following this research (i.e. housed for use in further research, etc.)

***Re: After the experiment, cows were housed for further studies.***

5. Accession number for RNAseq data:

***Re: We added in method section***

“ Raw sequencing reads of circulating miRNAs (publicly available on the GEO database accession number GSE150912)…” <https://www.ncbi.nlm.nih.gov/geo/query/acc.cgi?acc=GSE150912>

Reviewer #1:
**C1**: Please explain in more detail: how many blood samples have been taken from the cows? Was there only one measurement per cow in spring and one in summer? Please specify when measurements were taken- what month and day were chosen?

***Re: we added in more detail as follow (pls see underlines)***

***“The all cows were kept inside the barn that was opened for natural ventilation. We calculated daily THI using the recorded air temperature and humidity, and chose the sampling date when daily minimum THI > 72 and daily maximum THI <72 lasted for more than four weeks (Fig 1). Whole blood was collected separately from jugular vein of the same cows (n=9) at two different environmental seasons (summer and autumn) using PAXgene Blood RNA tube (2.5 ml/cow; Qiagen, 762165, California, USA) and vacutainer tube containing sodium heparin (10 ml/cow; BD, vacutainer®, 367874, Franklin Lakes, NJ, USA). PAXgene Blood RNA tubes were stored at -80℃ until miRNA extraction. Heparin tubes were immediately centrifuged at 3,000×g for 10 min at 4℃. Extracted plasma was stored at -80℃ until progesterone assay.”***

***The rectal temp. was measured with three technical replications when the blood was sampled as described in the method section, but other parameters such as body weight, milk yield, and ambient temperature/humidity were measured and recorded daily using automatic measuring instruments as described in the method section.***

***In Fig 1, you may find the date of sampling and information about THI; 15^th^ Aug, and 17^th^ Oct.***

C2: As parameter of exposure to heat was chosen THI in barn. Were cows at any time exposed to the outside temperatures?

***Re: Yes, the barn was opened to outside environment, but measuring devices were located inside the barn***

C3: Pregnancy as a matter of investigation: were all pregnant cows of the same gestational age?

***Re: They were not exactly the same age from two and a half months to three months when the blood samples were collected.***

***Pls see specific info. in the M&M section; All cows had 227±45.5 (mean±standard deviation) average milking days (Individual cow records including age, parity, and calving date in S1 Table).***

C4: In the present model of investigation where comparison of blood samples of the same pregnant cows but in different time period (and different gestational age) were made in order to investigate factor-heat stress could be disputable. Namely, differences observed in different time periods could be related to pregnancy state and not heat stress. Differences in progesterone levels observed also, could be related to the stage of pregnancy and not necessary induced by heat stress.

***Re: we added in the discussion section as follow***

***“As described above, the blood samples were collected from the same animals including pregnant and non-pregnant cows, suggesting that the different period or gestational age may affect the profile of gene expression. Thus, we first identified differential expressed genes in both pregnant and non-pregnant cows under HS, reflecting effects of HS and different time periods including different stage of lactation. However, the effects may be canceled out by the common genes both pregnant and non-pregnant cows, and their parities with different stages of lactation (S1 Table). In addition, profiles of putative target genes by DE miRNAs are also associated with metabolism and reproductive system, supporting that HS may induced the DE miRNAs.”***

We agree with your comments regarding progesterone, so we deleted some sentence in abstract, but left in the main text because our findings (elevated P4 in pregnant in HS) means pregnant cows are more susceptible to HS.

***“ ~~Particularly, progesterone concentrations known to have maternal warming effects were at similar levels in non-pregnant cows but significantly increased in pregnant cows under heat stress conditions~~…***

***~~with elevated progesterone concentrations~~”***

C5: Line 105: the authors stated: “Except for two cows, all cows were similar…” Were these differences taken into account considering results analysis?

***Re: No, we deleted this sentence.***

C6: Results: Statistical significances were not labeled neither in Table 1 nor in Figures

***Re: we labelled in Table 1 and figures***

C7: Line 224: it is not stated under what conditions significance was observed

***Re: we added “under HS conditions)”
“the rectal temperatures of pregnant cows (40.15℃±0.17) were higher than that of non-pregnant cows (39.36℃±0.1, P < 0.05, Table 1) under HS conditions”***

C8: Line 237: please rephrase, it is not clear at what time point significant difference was observed and please explain …”and milk increased after HS”? compared to what? That’s not what Figure shows.

***Re: We rephrased to clarify our findings as follow***

***“Relative average milk yield (AMY) decreased gradually in both pregnant and non-pregnant cows, compared to milk yield of May, particularly a significant reduction was observed in pregnant cows during extremely hot summer (August), and the recovery of AMY was observed in September (Fig 2A), indicating HS more severely may affect milk production of pregnant cows”***

Reviewer #2: Comments to Authors:
Major points
C1: The authors do not seem to statistically analyze the large data set of RNA-seq appropriately, as shown in Line 185-186. For the large data set analysis, the authors should do multiple comparison and test the significance of the difference between the treatments by FDR, Bonferroni, or Benjamini criteria to avoid detection of false positives. They just describe ‘P < 0.05’, but do not mention the statistical analysis. This is of critical importance, which further affects not only subsequent screening and PCR of DE miRNAs, but also bioinformatics analyses. Using the incorrectly processed data, they will fail to reach correct appropriate conclusion and interpretation of the data, which will mislead the readers of this manuscript.

***Re: As reviewer pointed out, a proper statistical analysis would be very important for large dataset such as RNAseq comparison. Moreover, multiple comparison (testing) should have done using FDR, Bonferroni etc. However, test significant by FDR and Bonferroni would be too much conservatives for such a small dataset like our samples. Furthermore, test significance would not tell biological significant. Therefore, we set a significant threshold 0.01.***

***We also described statistical procedure in the manuscript (Material and Methods) as below, In addition, our qRT-PCR validate our analysis of RNAseq data***

***“The differentially expressed gene analysis was performed by Limma-voom v3.34.9 R package (Smyth, 2005; Law et al., 2014; Ritchie et al., 2015). Before the analysis, genes which had raw read counts were filtered out by ‘filterByExpr’ function in edgeR R package (Robinson et al., 2010). Furthermore, TMM normalization was performed to normalize each library size using ‘calcNormFactors’ function in edgeR. Next, we used ‘voom’ function of Limma for read counts to transform to logarithmic (base 2) scale prior to linear modeling. Finally, empirical Bayes and moderated t-test were used to detect differentially expressed genes between two groups. The threshold to identify differentially expressed genes was set up to the p-value < 0.01 and the logarithm fold change |logFC| > 2.”***

C2: Furthermore, the R-package for methods to visualize DE miRNAs is not provided.

***Re: edgeR was used for differential expression (DE) analysis in R language. Pls see our response above***

C3:In addition, comparison of the treatments is not described, for example, no animal numbers for each treatment.

***Re: we added them in the experimental animal and blood collection (sub-section of material and methods);***

***“Pregnant (n=4) and non-pregnant (n=5) cows were used”***

***And sampling were performed two times (summer and autumn) “when daily minimum THI > 72 and daily maximum THI <72 lasted for more than four weeks (Fig 1)”***

Minor points
C4: Line 41: Define miRNA.

**Re: We spelled out; microRNAs (miRNAs)**

C5: Line 44: It is obvious that circulating miRNAs do not directly regulate gene expression in the cells of the target tissues. How do the authors explain the action of circulating miRNAs on these target cells?

***Re: Admittedly, we do not have direct evidence for the gene regulation by miRNA, but our findings of differential miRNA expression as biomarkers may be involved in or associated with the ovarian function under the heat stress. Therefore, we tried to explain possible function of DE miRNA to biological function. In addition, we can find the similar studies (Pregnancy-Ioannidis and Donadeu 2016; Heat stress-Zhung et al., 2014)***

C6:Line 67: Holstein Friesian ?

***Re: Yes, we corrected to Holstein Friesian***

C7: Line 91: Define miRNA.

***Re: Yes, we corrected to microRNA(miRNA)***

C8: Line 104-105: the sentence ‘four of them were pregnant …..3.25.’ does not make sense.

***Re: we deleted and added (individual cow records in S1 Table)***

C9: Line 141: Define (spell out) NHS.

***Re: we checked and spell out;***

***“under HS (Heat Stress; THI: 86.29) and NHS conditions (Non-Heat Stress; THI: 60.87)” in Rectal temperature in method section. .***

C10: Line 163: Show the reason why the authors chose miR-128 for endogenous control.

***Re: We refer to Ioannidis and Donadeu’s study “Changes in circulating microRNA levels can be identified as early as day 8 of pregnancy in cattle”; miR-126 was least variable. We cited this paper in the method section.***
