# Supplementary material for: Analysis of circulating-microRNA expression in lactating Holstein cows under summer heat stress
Source: PLoS One. 2020 Aug 31;15(8):e0231125. doi: 10.1371/journal.pone.0231125 (PMC7458322; doi:10.1371/journal.pone.0231125)
Supplement: Attachment — Submitted filename: Response to reviewers July.docx [file pone.0231125.s005.docx]

Reviewer #1:

C1 :Although improved, some methodological issues remain unclear.
Namely, study results showing different progesteron levels in the same cow with months apart could not be attributed to heat stress only, since progesteron levels show oscilations during pregnancy (Stabenfeldt et al, 1970). Therefore, this should be stated along with other observations related heat stress and pregnancy.

***Re: We delete materials & methods, results and discussion section about progesterone.***

C2: In Discussion, line 402: an adjective “reliable“ should be omitted since the present study did not show that. For such conclusion, larger datasets as well as adequate statistical analyses to detect accuracy of miRs as biomarkers are needed.

***Re: We deleted and revised (pls refer to our response to C5)***

***“ the selected miRNA could be potentially used to determine response to HS”***

C3: All in all, limitations of the present study need to be pointed out: small datasets, study design and statistical approach

Re: we added in conclusion section.

***“Admittedly, the our findings of the present study are limited by the small datasets , their statistical approach, experimental designs that could be attributed to the inherent limitations of the samples (synchronized gestation ages, parities, lactation stages) and heat stress condition (frequent, intense, and duration). Thus, we need to verify our results using model animals or the climate control system (temperature and humidity)”.***

Reviewer #2:

C4: Line 40-42, 382-385: These sentences should be revised or deleted. The authors mention, "we found that differentially targeted progesterone biosynthesis (StAR) and the function of corpus luteum-related genes (CCL11, XCL)", however, they only predicted the targeted genes by use of bioinformatic databases but did not obtain any evidence showing that the miRNAs target and regulate the potential target genes. In addition, as for almost all the circulating miRNAs, it is difficult to determine which tissues release circulating miRNAs and where they are up-taken. I think, therefore, relationships between circulating miRNAs and the target genes in potential target tissues cannot be easily concluded without any data regarding gene expression in the target tissues.

***Re: we deleted line 38-42, and revised. Pls see our response to C5, We mentioned predicted genes/function via bioinformatics analysis***

C5: Line 368-410: Regarding the potential target genes, these paragraphs are with too much description only for speculation from the predicted data, and therefore the volume of the description should be much more reduced. If the authors show transcriptomic data, then they could discuss more about the target genes and the physiological adaptation, however, actually this is not the case here. For this reason, the line 409-410 should be deleted because the authors did not show whether the signaling pathways were responsible for several important regulations in lactating cows under HS conditions or not in fact.

***Re: We rewrote the following paragraphs to achieve a more concise description.***

***Integrative approaches using the predicted target genes of DE miRNAs, and DAVID identified several KEGG pathways such as FoxO signaling, actin cytoskeleton, Rap1 and rapamycin (mTOR) signaling that are involved in transcription activation of heat shock proteins, mitochondria distribution and activity, regulation of energy homeostasis including glucose, and lipid metabolism [49-57]. We also found, in line with a previous study [25], immune response related pathways such as chemokine and TNF signaling [58, 59]. In addition, several DE miRNAs (bta-miR-146b, bta-miR-20b, bta-miR-29d-3p, bta-miR-1246) were differentially expressed in summer heat-stressed pregnant cows. Interestingly, bioinformatics analysis showed their predicted target genes are associated with function of corpus luteum including progesterone biosynthesis [60-61] and prolactin signaling pathways (Table S4).***

***----------------------------------------------------------------------------------------------------------------------------------------------------- Comprehensively, our findings suggest that the experimentally verified miRNA targets and in silico analysis reflect that the selected miRNA could be potentially used to determine response to HS.***

***Conclusions***

***We analyzed physiological HS indicators such as milk yield, rectal temperature, ADG, in pregnant and non-pregnant cows and found that pregnant cows are more vulnerable under HS conditions. In transcriptome analysis of miRNAs using RNA-sequencing, 11 miRNAs (bta-miR-19a, bta-miR-19b, bta-miR-30a-5p, several from bta-miR-2284 family) were differentially expressed (|FC| ≥ 2, P < 0.05) in both pregnant and non-pregnant cows. In pregnant cows under HS, two miRNAs (bta-miR-20b, bta-miR-29d-3p) were upregulated while two miRNAs (Bta-miR-146b, bta-miR-1246) were downregulated. These selected miRNAs could be potential biomarkers associated with HS. Admittedly, the our findings of the present study are limited by the small datasets , their statistical approach, experimental designs that could be attributed to the inherent limitations of the samples (synchronized gestation ages, parities, lactation stages) and heat stress condition (frequent, intense, and duration). Thus, we need to verify our results using model animals or the climate control system (temperature and humidity).***
